# Characteristics of Settling Coral Reef Fish Are Related to Recruitment Timing and Success

**DOI:** 10.1371/journal.pone.0108871

**Published:** 2014-09-24

**Authors:** Tauna L. Rankin, Su Sponaugle

**Affiliations:** 1 Division of Marine Biology and Fisheries, Rosenstiel School of Marine and Atmospheric Science, University of Miami, Miami, Florida, United States of America; 2 NOAA National Marine Fisheries Service, Office of Habitat Conservation, Coral Reef Conservation Program, Silver Spring, Maryland, United States of America; 3 Department of Integrative Biology, Oregon State University, Hatfield Marine Science Center, Newport, Oregon, United States of America; Leibniz Center for Tropical Marine Ecology, Germany

## Abstract

Many marine populations exhibit high variability in the recruitment of young into the population. While environmental cycles and oceanography explain some patterns of replenishment, the role of other growth-related processes in influencing settlement and recruitment is less clear. Examination of a 65-mo. time series of recruitment of a common coral reef fish, *Stegastes partitus*, to the reefs of the upper Florida Keys revealed that during peak recruitment months, settlement stage larvae arriving during dark lunar phases grew faster as larvae and were larger at settlement compared to those settling during the light lunar phases. However, the strength and direction of early trait-mediated selective mortality also varied by settlement lunar phase such that the early life history traits of 2–4 week old recruit survivors that settled across the lunar cycle converged to more similar values. Similarly, within peak settlement periods, early life history traits of settling larvae and selective mortality of recruits varied by the magnitude of the settlement event: larvae settling in larger events had longer PLDs and consequently were larger at settlement than those settling in smaller pulses. Traits also varied by recruitment habitat: recruits surviving in live coral habitat (vs rubble) or areas with higher densities of adult conspecifics were those that were larger at settlement. Reef habitats, especially those with high densities of territorial conspecifics, are more challenging habitats for young fish to occupy and small settlers (due to lower larval growth and/or shorter PLDs) to these habitats have a lower chance of survival than they do in rubble habitats. Settling reef fish are not all equal and the time and location of settlement influences the likelihood that individuals will survive to contribute to the population.

## Introduction

Understanding the mechanisms that drive variability in population dynamics is a fundamental goal of ecology. Most marine organisms have complex life cycles with multiple ontogenetic shifts, and processes occurring during the early life stages can influence population demography of later stages. For taxa such as coral reef fishes, which are demersal as juveniles and adults but possess a pelagic larval phase, population replenishment can be highly variable in space and time (reviewed in [1]) and patterns in settlement and post-settlement processes can strongly influence population size and distribution [2]–[4].

Common causes of temporal and spatial variability in settlement and recruitment range from environmental processes (e.g., reproductive seasonality related to water temperature [5], [6]; spawning and settlement synchrony to lunar and tidal amplitude cycles [7], [8]), and transport of larvae by climatological or oceanographic processes [8]-[11], to location and quality of settlement habitat [12], and distribution and composition of reef residents (e.g., predators, competitors [13]). Recently, more attention has focused on the characteristics of settlers and how variation in the quality of settlers may contribute to observed patterns in settlement and recruitment.

Early life history traits, such as larval growth and size-at-settlement, vary in space and time and have been linked to settlement magnitude [14]–[17]. Larval encounter with particular water temperatures, water masses, or oceanographic features can influence the early life history traits of settlers [18]–[21]. Further, these traits can carry over to affect post-settlement survival and recruitment success [22]–[24]. For many reef fish species, faster larval growth, shorter larval duration, larger size-at settlement, or higher condition-at-settlement [20], [22]–[30] confers a survival advantage to new recruits. Such trait-mediated selective mortality influences the composition of young entering the benthic population.

The magnitude of benthic mortality also likely varies with timing and location of settlement. For instance, many species of reef fish settle during the darkest half of the lunar cycle [7], [8], [31], [32], potentially due to reduced predation by visual predators during this time [33]. While the presence of conspecifics can enhance post-settlement survival in some species [34], high densities of conspecifics also can increase the mortality of new recruits [28]. Habitats also vary in the abundance and composition of predators [35], [36] and refuges from such predation [37]. Thus, spatially and temporally variable predation pressure may interact with the variable traits of incoming settlers [38], [39] and substantially complicate patterns of selective mortality of recruits.

Here, we examined spatial and temporal variation in settlement and recruitment for a common coral reef fish and tested whether this variation was associated with certain growth-related early life history traits. Specifically, we explored whether the early life history traits of settling larvae varied with the lunar timing of settlement and recruitment habitat, including density of conspecifics, and how the trait composition of juveniles changed over time.

## Materials and Methods

### Ethics Statement

All fish were collected and handled in accordance with the University of Miami Animal Care and Use Committee regulations as approved under UM-ACUC Permits 01–056ad and renewal, 05–019ad, 07–068ad01 and renewal02–056. Field collections were authorized under permits 00S–524 and 02R–524A, 04SR–524, 07SR–1032A from the Florida Fish and Wildlife Conservation Commission, and permits 2001–004, 2002–025A, 2004–024 from the Florida Keys National Marine Sanctuary.

### Study site and physical data sampling

The seasonally variable Florida Current dominates the circulation of the Florida Keys. Cyclonic eddies propagate along the frontal boundary of the Florida Current and can transport larvae to nearshore reefs [10]. Mean water temperature in the Florida Keys varies seasonally by approximately 10°C and can significantly influence variation in reef fish early life history traits and recruitment strength [19], [24]. Thus, to characterize the inshore environment of the Florida Keys and control for temperature effects, daily water temperatures were obtained from the National Underwater Research Center, which continuously records water temperature at 21 m depth at Conch Reef in the upper Florida Keys (24°59′N, 80°25′W).

### Biological sampling

The bicolor damselfish *Stegastes partitus* is common throughout the Caribbean. Adults are highly territorial and spawn demersal eggs on a monthly basis throughout the year [31], [40], after which, the males guard the embryos for ∼4 d until hatching [31]. Larvae spend a mean of 30 d in the plankton before settling to the reef and metamorphosing into juveniles overnight [32]. Juveniles and adults maintain benthic territories on spur and groove reefs and are found in highest densities in areas of high current flow and within dead coral rubble piles [37], [41]. The timing of settlement appears to be synchronized with lunar phase, with pulses occurring during the third quarter and/or new moon [8], [31], [32]. Seasonal peaks in *S. partitus* settlement to the Florida Keys typically occur during summer months [8], [42]. *Stegastes partitus* is a useful model species for studying processes affecting the early life history of coral reef fishes because it is widely distributed, is integral to the trophic dynamics of the reef community [43], [44], is easy to observe and collect at most life stages, and has otoliths (ear stones) that provide a daily record of events occurring during early life.

Newly recruited *S. partitus* juveniles were surveyed monthly on six different reefs within the Florida Keys National Marine Sanctuary offshore of Key Largo, Florida, for 65 mo. over 6 yrs from April 2003 until August 2008. To ensure that each survey captured all of the settlement for a given month, surveys were timed to coincide with the full moon, which is known to be a period of low settlement. Four of the reefs are part of the bank reef tract ∼10 km offshore: French Reef (FR; 25°02.06′N, 80°21.00′W), Sand Island Reef (SI; 25°01.09′N, 80°22.08′W), Molasses Reef (MO; 25°00.74′N, 80°22.40′W), and Pickles Reef (PI; 24°59.23′N, 80°24.88′ W), two of which are sanctuary protected areas (MO, and FR; see Fig. S1 for map). The other two sites, Triangles (TR; 25°01.153′N, 80°26.272′W) and White Banks (WB; 25°02.609′N, 80°22.133′W), are inshore patch reefs. Recruit density was estimated for two habitats within the bank reef sites, the reef itself and dead coral rubble at the base of the reef, by counting the number of newly recruited juveniles within 15 haphazardly placed 5×1 m transects in each habitat. Within the two inshore patch reefs (TR and WB), only reef habitat was surveyed due to the lack of coral rubble habitats surrounding the reef. At SI, PI, TR, and WB, all censused recruits were collected by divers using hand nets and the anesthetic Quinaldine. Because FR and MO are Sanctuary Protected Areas, benthic sampling was not permitted. During 2007 and 2008, after each survey, additional collections were made off of the transects at the non-sanctuary protected sites to supplement sample sizes. All collected juveniles were immediately stored in 95% EtOH following collection to preserve their otoliths.

We also examined settlement-stage *S. partitus* larvae collected in larval light traps at FR, SI, MO, and PI from June 2001 to January 2004 as part of two separate studies [8], [10] to compare differences in growth-related traits and magnitude of supply with the data from recruits. Three replicate light traps were deployed every other night at each of 2–3 sites for ∼3 summer months in 2001, 6 summer months in each of 2002 and 2004, and twice a month for 6 winter months in each of 2003 and 2004.

### Otolith analysis

Prior to dissection, the standard length (SL) of each specimen (n = 1291; 465 larvae and 826 juveniles) was measured digitally to the nearest 0.01 mm. The otoliths were extracted using standard dissecting techniques and placed in a drop of medium viscosity immersion oil on a microscope slide to clear for a minimum of 30 d. Based on ease of reading, only the lapilli were examined [32]. The clearest lapillus was chosen from each individual and viewed under 400× magnification through a Leica DMLB microscope equipped with a polarized filter between the first stage and light source. The image was captured by a Dage MTI video camera and analyzed using Image Pro Plus 4.5 software (Media Cybernetics). Each otolith was read once blind (i.e., without sample information available) and saved as a digital file. Every 5^th^ individual was measured a second time (sensu [45]). The images of the remaining 1029 fish were examined to determine if there was any ambiguity in the placement of the increments and an additional 161 fish were aged a second time, resulting in a total of 418 fish aged twice. Otoliths were rejected where the difference between the first and second reads was >5%, resulting in six exclusions. Otolith analysis was utilized to determine the following early life history traits: post-settlement age (number of concentric increments after the settlement mark), pelagic larval duration (PLD; number of concentric increments from the primordium to the settlement mark), larval and juvenile growth rates (widths between consecutive increments), and size-at-age (otolith radius-at-each age, including settlement).

### Data analysis

#### Interannual and monthly variation in settlement and recruitment magnitude

Otolith analysis was used to back-calculate date of hatching (collection date minus PLD and post-settlement age) and date of settlement (collection date minus post-settlement age). Spawning dates were calculated by subtracting 4 d from the hatch dates, for average egg development [31]. Daily settlement magnitude was estimated from the monthly measures of recruit density coupled with the settlement dates of the collected fish and adjusted for estimated mortality with age (sensu [46]). Mortality was estimated by fitting a function (equation: y = 0.0671x^2^–5.0165x+89.763; where x  =  juvenile age in days, and y  =  number of recruits) to the age frequency distribution of all 9+ d old recruits (Fig. S2). For this calculation only these older recruits were used because the youngest fish were underrepresented in the samples due to the timing of sampling (during the low settlement period around the full moon) and the fact that older recruits do not shelter as much. The mortality function was then used to adjust the number of settlers per day based on the age of the recruits that settled on that day. Note that where recruitment densities from surveys were used in an analysis directly, we use the term “recruitment”; where recruit densities were adjusted for post-settlement mortality, we use the term “settlement.” For larvae collected in light traps, we use the phrase “larval supply.”

#### Lunar and tidal periodicity in settlement and spawning

A lunar day of settlement was assigned for each aged fish (day 1 corresponding to the new moon; day 8 corresponding to the first quarter; day 15 corresponding to the full moon; day 23 corresponding to the third quarter). Each fish was also assigned a tidal amplitude day (day 1 corresponding to the maximum tidal amplitude closest to the new moon). These data were collapsed into a single lunar and maximum tidal amplitude cycle. Rayleigh tests were utilized to determine if light trap catches or settlement magnitude were uniformly distributed across the lunar or tidal amplitude cycles [47]. When data were non-randomly distributed, the mean lunar or tidal amplitude day about which the data were centered was calculated.

#### Variation in early life history traits by lunar phase of settlement and over time

Aged fish were divided into three age groups: larvae (all settlement-stage larvae collected from 2001–2004 light traps), newly settled recruits (1–10 d old), and juveniles (11–28 d old). To determine if the composition of early life history traits varied with lunar timing of settlement, otolith derived traits were compared among lunar phases by age group using Kruskal-Wallis procedures (SYSTAT version 11.0; [48]), followed by post-hoc non-parametric multiple comparison tests [47]. For this and all subsequent analyses, individuals were classified into four groups by lunar phase, which included each lunar phase and 3–4 d on either side of the phase (i.e., first quarter moon: days 5–11, full moon: days 12–18, third quarter moon: days 19–26, new moon: 27–4). To determine whether patterns of selective mortality varied with lunar phase, we compared the traits among age groups by lunar phase in the same manner. Directional selection on a given trait was identified where there was a shift in the distribution of that trait in the older juveniles compared to younger recruits or larvae, and between recruits and larvae. Previous results demonstrated that temperature influences the intensity and direction of selective mortality acting on some *S. partitus* early life history traits (i.e., larval growth and PLD; [24]). In other species, such traits can influence recruitment magnitude [14], [15]. To control for the effect of temperature we divided monthly cohorts into three categories by temperature (<25°C, 25 to <28°C, and >28°), which also corresponded roughly with season. Over 65% of the recruits collected were from the warmest temperature group and therefore comparisons of traits with other processes (i.e., settlement magnitude, lunar phase of settlement, location of settlement, and age group) were confined to individuals that settled during the warmest months.

#### Variation in early life history traits with settlement magnitude

Early life history traits of juveniles were more variable during smaller settlement events than larger ones, which precluded a significant linear relationship between monthly settlement magnitude and any of the individual traits. Thus, to determine whether the composition of growth-related traits varied with settlement magnitude and changed over time, the estimated total number of settlers was divided into two groups: those that settled in small events where monthly settlement was ≤10% of the seasonal total, and those that settled in large events where monthly settlement was >10% of the seasonal total. The 10% criterion was selected a posteriori based on the distribution of the data. Early life history traits were compared between large and small events using the Mann-Whitney U test (SYSTAT version 11.0; [48]). To control for the effects of temperature and lunar phase of settlement, comparisons were limited to individuals that settled during the darker half of the lunar cycle, from cohorts that encountered mean water temperatures of >28°C. Comparisons examining mean juvenile growth over the first two d post-settlement were limited to recruits that were at least 2 d old (2–10 d old versus 11–28 d old).

#### Variation in early life history traits by settlement habitat

Variable habitat quality (i.e., food abundance and quality, substrate suitability) and habitat-specific differential predation pressure may lead to preferential habitat selection by larvae and/or differential survival of recruits [49]. To examine whether early life history traits varied by microhabitat choice, traits were examined for recruits in reef and rubble habitats using the Mann-Whitney U test (SYSTAT version 11.0; [48]). To control for the influence of settlement magnitude on early life history traits, we compared the traits between reef crest and rubble habitats only for the warmest temperatures when recruitment pulses were relatively large.

#### Influence of older conspecifics on recruitment magnitude and early life history traits

The density of conspecifics may make an area more or less favorable for larval settlement or juvenile survival; therefore recruit density was also regressed against juvenile (20–30 mm SL), intermediate (30–50 mm SL), and adult (>50 mm SL) conspecific densities to determine if recruitment magnitude was related to densities of conspecifics. Growth-related traits were also regressed against conspecific density to determine if composition of traits was related to the presence of juvenile, intermediate, or adult conspecifics.

## Results

### Interannual and monthly variation in settlement and recruitment magnitude

**Figure 1:**
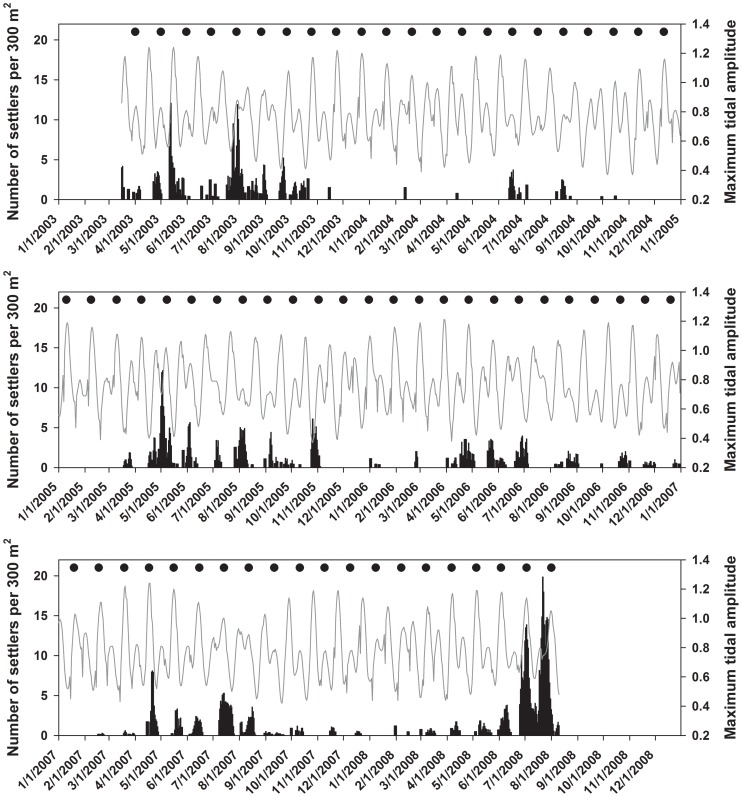
Daily back-calculated settlement of *Stegastes partitus* in reef and rubble habitat at Pickles and Sand Island Reefs combined (bars). Timing of settlement of collected recruits was adjusted for monthly density estimates and estimated post-settlement mortality. Maximum tidal amplitude is indicated by a line, and new moons by solid circles. Asterisks indicate months when sampling did not take place.

Over six years, *Stegastes partitus* settlement varied on both an interannual and monthly basis (Fig. 1). Annual *S. partitus* settlement was similar for most years, with the exception of 2004, which was three-fold to over six-fold less than the other five years, and 2008, which had the highest magnitude. The highest mean recruitment across years occurred during July and August (Table 1). The coefficients of variation (CV) were relatively high for all months, indicating high interannual variability, but July and August were on the lower end of the spectrum (Table 1). February had the lowest mean recruitment and largest CV across years.

**Table 1:** Monthly variability in recruitment of *Stegastes partitus* to reefs in the upper Florida Keys.

Month	Mean number of recruits (#300 m^−2^)	SD	CV
January	0.31	0.79	2.53
February	0.17	0.73	4.23
March	0.29	0.80	2.72
April	1.70	3.11	1.83
May	3.39	7.00	2.07
June	4.55	6.91	1.52
July	5.39	8.58	1.59
August	6.57	9.73	1.48
September	2.64	3.16	1.20
October	1.19	2.66	2.23
November	2.02	3.21	1.59
December	0.25	0.61	2.43

New recruits were surveyed monthly by divers for 65 months from April 2003 to August 2008.

### Spatial patterns in recruitment magnitude

Recruitment of *S. partitus* to the offshore bank reefs was almost 12-fold higher than to the inshore patch reefs. When recruitment to the rubble habitats around the base of the reefs was included, the difference increased to 29-fold, because there was high recruitment to the rubble surrounding the offshore reefs, but no recruitment to the seagrass surrounding the inshore patch reefs. Within the offshore reefs, recruitment was three-fold greater in the rubble habitat compared the reef habitat.

### Lunar and tidal periodicity in settlement and spawning

Within months, back-calculated settlement was strongly periodic (Fig. 1). For the aged individuals, there was a significant peak in settlement on day 2 of the lunar cycle (one day after the new moon; Table 2). After adjusting for recruitment magnitude (density) and post-settlement mortality, the peak in settlement shifted to day 29 (one day before the new moon) as there was greater representation of juveniles that settled around the third quarter moon (days 19–26; Fig. S3). This pattern corresponded closely to light trap catches of settlement-stage larvae from 2003–2004, where the mean peak in settlement was calculated to be on day 27 (Table 2). The maximum tidal amplitude cycle is tightly coupled with the lunar cycle in Florida, but specific timing of maximum amplitude tides with lunar phase shifts seasonally (Fig. 1). Settlement of juveniles corrected for post-settlement mortality peaked on day 1 of the maximum tidal amplitude cycle (Table 2, Fig. S3).

**Table 2:** Rayleigh statistics for lunar and tidal amplitude synchrony in settlement and successful spawning of *Stegastes partitus* in the upper Florida Keys.

	n	Z	Day	s
**Lunar settlement**				
Aged recruits	786	134.5	2	5.0
Recruits adj. for mortality	1429	142.0	29	5.4
Settlement-stage larvae	415	64.1	27	5.1
**Tidal amplitude settlement**				
Recruits adj. for mortality	1383	161.6	1	5.3
**Lunar spawning**				
Aged recruits	788	89.0	1	5.3
Recruits adj. for mortality	1433	86.4	26	5.7
Settlement-stage larvae	196	21.7	21	5.3

Recruits were obtained from monthly surveys; settlement-stage larvae were obtained from light traps. Otolith microstructure of recruits was used to back-calculate settlement timing and recruit densities were adjusted for mortality to provide a more precise estimate of settlement magnitude. All tests were significant at p<0.001. Z is the Rayleigh test statistic; Day is the mean peak in lunar or maximum tidal amplitude calculated from mean vector angle; s is the mean angular deviation; Day 1 is the new moon.

Back-calculation of spawning day for aged juveniles revealed a peak in successful spawning on day 1 of the lunar cycle, corresponding to the new moon (Table 2, Fig. S4). Using the adjusted values of settlement intensity as above, the distribution remained non-random with a mean peak in spawning on day 26, several days closer to the spawning distribution back-calculated from the light-trap larvae which peaked on day 20 (Table 2, Fig. S4).

### Variation in traits by lunar phase of settlement

**Figure 2:**
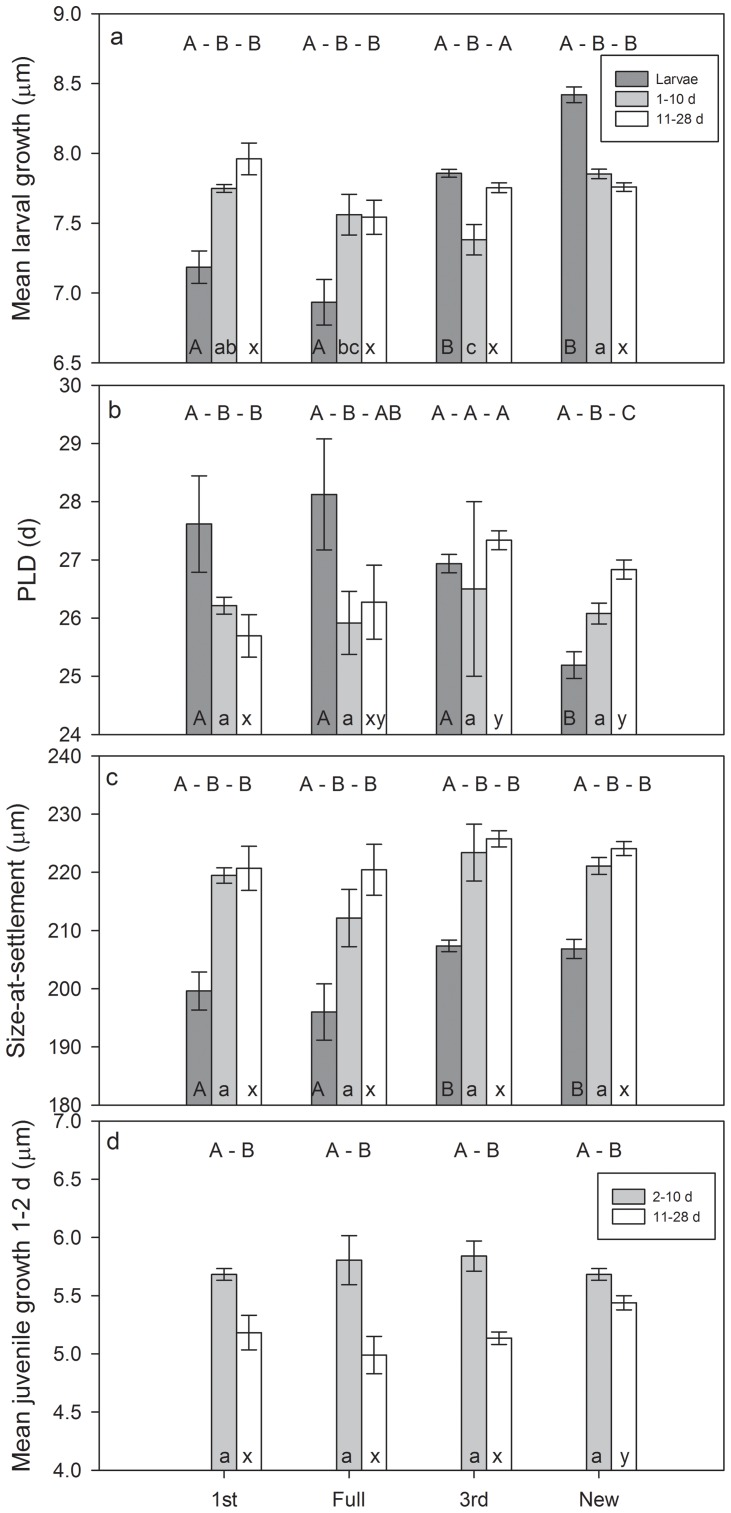
Mean otolith-derived (a) larval growth, (b) pelagic larval duration (PLD), (c) size-at-settlement, and (d) early (2 d) juvenile growth of *Stegastes partitus* settlement-stage larvae, recruits (1–10 d post settlement), and juveniles (11–28 d) by lunar phase of settlement. Effect of temperature was minimized by including only 25 monthly cohorts collected from May 2003 to August 2008 with mean water temperatures of >28°C. Error bars represent SE. Different letters above the bars indicate significant relationships among age groups, within lunar phases. Different letters within the bars indicate significant relationships among lunar phases, within age groups (capital A, B denote differences within larvae, lowercase a, b denote differences within 1–10 d old recruits, lowercase x, y denote differences within 11–28 d old juveniles). Settlement-stage larvae are not included in the last panel because they had not yet settled so did not exhibit juvenile growth.

We have previously shown that early life history traits in *S. partitus* are closely linked. PLD is inversely related to larval growth and both of these traits determine size-at-settlement, the most important trait influencing juvenile survivorship in *S. partitus* [24]. Fish that settle at large sizes have higher survivorship but also spend more time in territorial defense and thus experience slower early juvenile growth [24]. All of these trait relationships obtained from tracking cohorts over time are evident in the patterns obtained in the present study; only here the focus is on variation over the lunar cycle. Early life history traits varied among fish settling on different lunar phases for all three age groups of *S. partitus*. Larvae settling to the reef on the third quarter and new moons experienced significantly faster larval growth and were significantly larger at settlement than larvae arriving on the first and full moons (Table 3, Fig. 2). Furthermore, the new moon settlers spent significantly less time in the plankton than larvae settling on the other lunar phases (Table 3, Fig. 2). At 1–10 d post-settlement, recruits had fairly similar traits among the different lunar phases of settlement, with the exception that new moon settlers had significantly faster larval growth than third quarter and full moon settlers. By 11–28 d post-settlement, juveniles that settled on the third quarter and new moons had significantly longer PLDs than first quarter moon settlers, and new moon settlers had significantly faster early post-settlement growth than first, full, and third quarter moon settlers (Table 3, Fig. 2).

**Table 3:** Results of Kruskal-Wallis tests comparing *Stegastes partitus* early life history traits among different phases of lunar settlement (1 =  first quarter; F  =  full moon; 3 =  third quarter; N  =  new moon); and among three age groups (L  =  larvae; R  =  recruits 1–10 d post-settlement; J  =  juveniles 11–28 d post-settlement).

	Trait comparison across lunar phases						Trait comparison by age group							
	LARVAE		RECRUITS (1–10 d)		JUVENILES (11–28 d)		1ST		FULL		3RD		NEW	
TRAIT	*P*	post-hoc	*P*	post-hoc	*P*	post-hoc	*P*	post-hoc	*P*	post-hoc	*P*	post-hoc	*P*	post-hoc
**Larval growth**	<0.001	1, F <3, N	0.008	3<1, N; F <N	0.113		<0.001	L <R, J	0.015	L <R, J	0.044	L, J> R	<0.001	L> R, J
**PLD**	<0.001	1, F, 3> N	0.962		0.003	1<3, N	0.016	L> R, J	0.045	L <R	0.125		<0.001	L <R <J
**Size-at-settlement**	0.001	1, F <3, N	0.533		0.334		<0.001	L <R, J	0.016	L <R, J	<0.001	L <R, J	<0.001	L <R, J
**Juvenile growth 1–2 d**			0.844		0.001	1, F, 3 <N	0.009	R> J	0.025	R> J	0.004	R> J	0.001	R> J

Trait-related selective mortality across age groups was evident for most settlers, but the strength and direction of selection varied by lunar phase. First and full moon settlers experienced selection for faster larval growth, lower PLDs, larger settlement sizes, and slower early juvenile growth (Table 3, Fig. 2). For third quarter moon settlers, there was no selective mortality related to larval growth or PLD, but selection for larger settlement sizes and slower early juvenile growth. For new moon settlers, directional selection on larval growth and PLD was opposite to that for first and full moon settlers. While the direction of selective mortality related to the critical trait of size-at-settlement was consistent across all lunar phases, first quarter/full moon settlers experienced stronger selective mortality than third quarter/new moon settlers (Table 3, Fig. 2).

### Variation in traits with settlement magnitude

**Figure 3:**
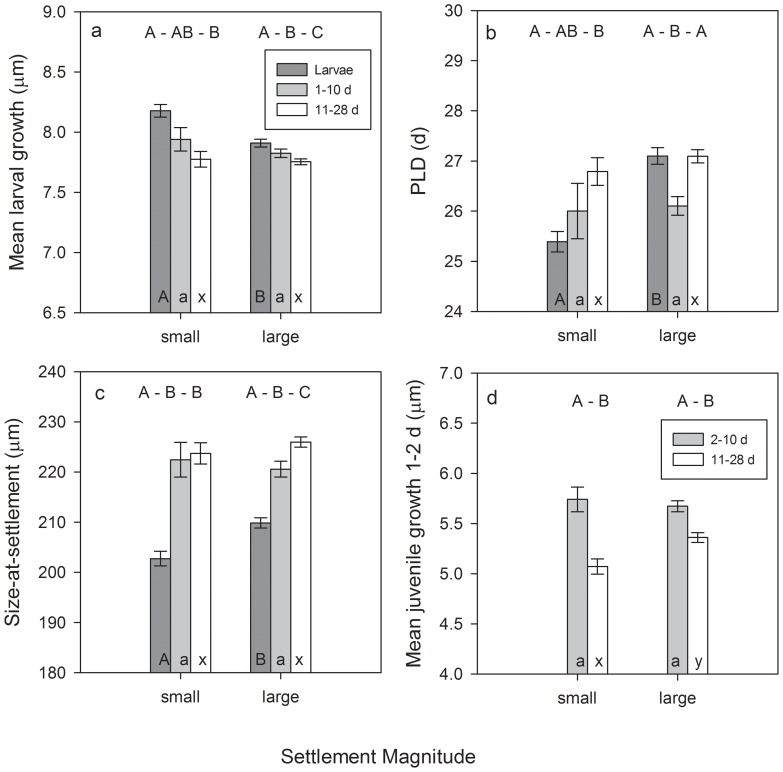
Mean otolith-derived (a) larval growth, (b) pelagic larval duration (PLD), (c) size-at-settlement, and (d) early (2 d) juvenile growth of *Stegastes partitus* settlement-stage larvae (dark gray bars), recruits (1–10 d post-settlement; light gray bars), and juveniles (11–28 d; white bars) by size of light trap pulse (larvae) and size of settlement event (recruits/juveniles). Effects of temperature and lunar phase were minimized by including only individuals from cohorts that encountered mean water temperatures of >28°C and settled during the darker half of the lunar cycle. Error bars are SE. Different letters above the bars indicate significant differences among age groups, within events of a given size. Different letters within the bars indicate significant differences between small and large events, within age groups (capital A, B denote differences within larvae, lowercase a, b denote differences within 1–10 d old recruits, lowercase x, y denote differences within 11–28 d old juveniles). Settlement-stage larvae are not included in the last panel because they had not yet settled so did not exhibit juvenile growth.

During peak settlement during the dark half of the lunar cycle between May-August, late stage larvae that settled to the reef in large events had significantly slower larval growth, longer PLDs, and larger sizes-at-settlement that those that arrived in small events (Table 4; Fig. 3). Selective mortality was evident for fish settling in both small and large events: there was selection for slower larval growth, shorter PLDs, and larger sizes-at-settlement (Table 4; Fig. 3). However, there were larger differences in traits between larvae and juveniles for fish that settled in small events, indicating stronger selective mortality pressure. Consequently, for older juveniles, there were few differences in traits between fish that settled in large vs. small events (p>0.05). However, juveniles that settled in large events had significantly faster early juvenile growth (p = 0.013; Fig. 3).

**Table 4:** Results of Mann-Whitney U tests comparing *Stegastes partitus* early life history traits between small and large settlement events for larvae, recruits (1–10 d post-settlement), and juveniles (1–28 d post-settlement); and among age groups (L =  larvae; R  =  recruits; J =  juveniles).

	Trait comparison by size of settlement event						Trait comparison by age group			
	LARVAE		RECRUITS (1–10 d)		JUVENILES (11–28 d)		SMALL		LARGE	
TRAIT	*P*	Relationship	*P*	Relationship	*P*	Relationship	*P*	post-hoc	*P*	post-hoc
**Larval growth**	<0.001	Small > large	0.550		0.857		0.023	L> J	<0.001	L> R> J
**PLD**	<0.001	small <large	0.210		0.220		0.038	L <J	<0.001	L, J> R
**Size-at-settlement**	<0.001	small <large	0.128		0.190		<0.001	L <R, J	<0.001	L <R <J
**Juvenile growth 1–2 d**			0.413		0.010	small <large	0.040	R> J	0.006	R> J

### Variation in early life history traits by settlement habitat & conspecific density

**Figure 4:**
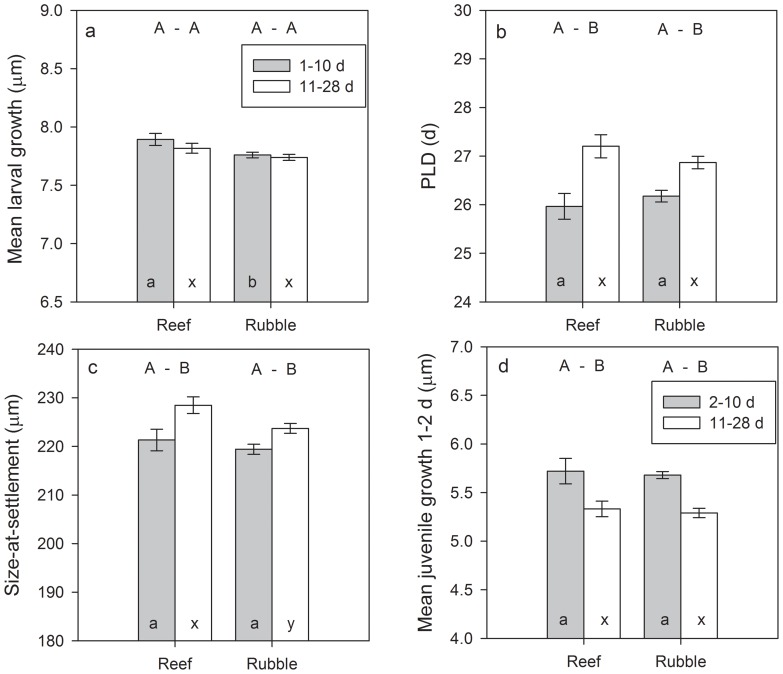
Mean otolith-derived (a) larval growth, (b) pelagic larval duration (PLD), (c) size-at-settlement, and (d) early (2 d) juvenile growth for two different age groups of *Stegastes partitus* recruits by habitat type (i.e., reef crest and rubble). Effect of temperature was minimized by including only 25 monthly cohorts collected from May 2003 to August 2008 that encountered mean water temperatures of >28°C. Error bars are SE. Different letters above the bars indicate significant relationships between habitats for a given age group. Different letters within the bars indicate significant relationships between age groups (selective mortality) by habitat (a, b denote differences between recruits (1–10 d post-settlement) and juveniles (11–28 d) in reef habitats, x, y denote differences between recruits and juveniles in rubble habitats.

Comparisons of mean traits among microhabitats for the two age groups of *S. partitus* revealed that there were generally few differences in traits among habitats. However, the trait most directly related to mortality, size-at-settlement, varied significantly between habitats in the oldest age group. While size-at-settlement did not differ between habitats for young recruits, over time, survivors (11–28 d old) in reef habitats were those that settled at larger sizes than those settling to rubble habitats (p = 0.018), suggesting that selective mortality is stronger in reef habitats. The only other trait varying between habitats was larval growth, which was significantly higher in young recruits (1–10 d old) in reef habitats (p = 0.030), but evened out between habitats over time (Fig. 4). General patterns of trait-based selective mortality between the age groups echoed those reported above and previously [24], however, this analysis by habitat reveals a larger shift in both PLD and size-at-settlement over time in the reef habitat, reflecting stronger selective pressures in that habitat.

**Figure 5:**
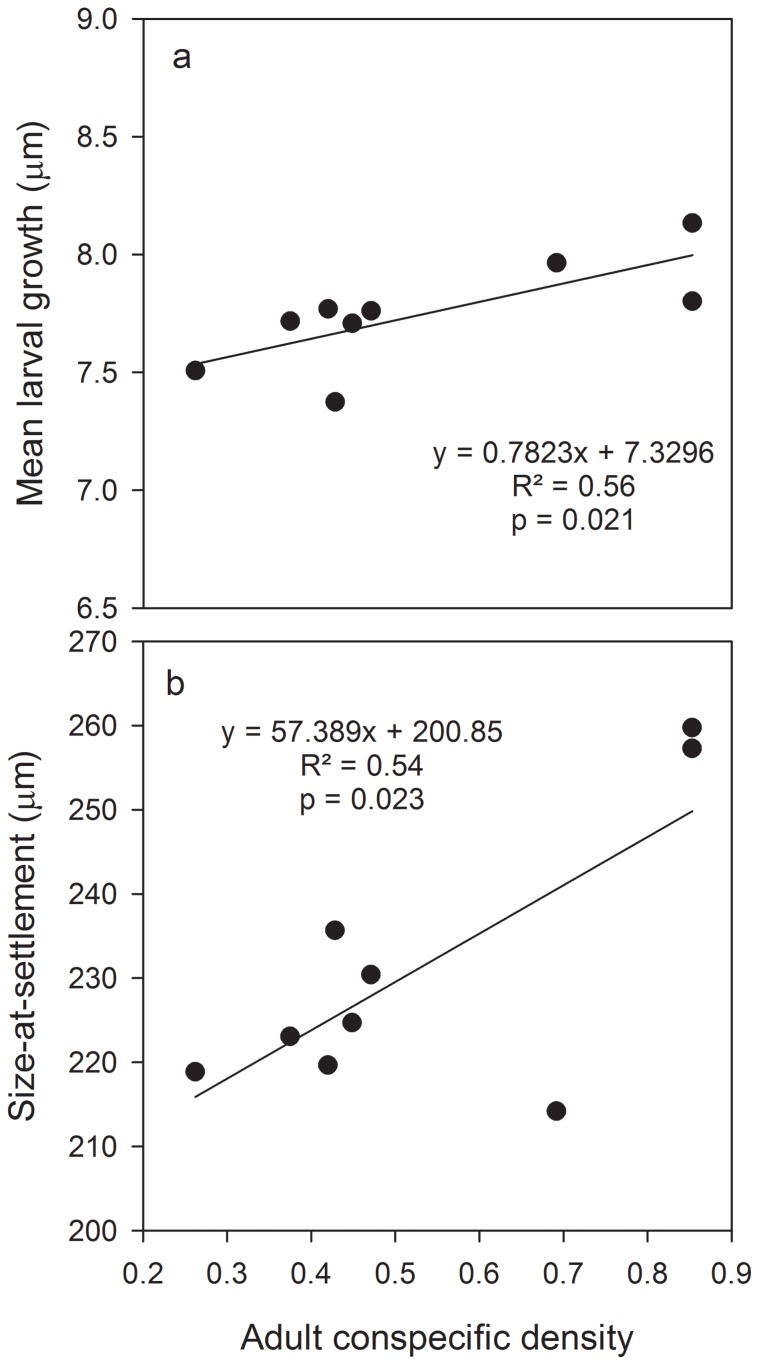
Relationship between mean otolith-derived (a) larval growth and (b) size-at-settlement of *Stegastes partitus* recruits and adult conspecific density from June 2007 to August 2008.

Considering the whole dataset, monthly recruit density was positively related to the density of >1 mo. old juvenile conspecifics (20–30 mm SL; R^2^ = 0.83, p<0.001). This positive relationship was maintained when the analysis was constrained to only cohorts settling during the warmest water temperatures (R^2^ = 0.45, p = 0.025). Recruit density was also positively related to the density of intermediate age conspecifics (30–50 mm SL; R^2^ = 0.042, p = 0.034), but there was no significant relationship between recruit density and adult conspecific density (p = 0.120). However, larval growth and size-at-settlement of recruits were positively related to adult density (Fig. 5). PLD and early juvenile growth of recruits were not significantly related to adult conspecific density (p>0.05).

## Discussion

Results of this nearly six year time series indicate that there is interannual, monthly (seasonal), and lunar periodicity in the settlement and recruitment of the common reef fish, *Stegastes partitus*, to the upper Florida Keys. Similar patterns have been demonstrated for *S. partitus* settling to other locations [7], [31], [32], as well as in a shorter study of larval supply in the Florida Keys [8]. In Panama, large pulses of *S. partitus* settlement were associated with faster early growth [14] and larger sizes-at-settlement [15]. Such traits not only influence recruitment magnitude, but can also affect juvenile survival of *S. partitus* [24], [28]. Here we tested whether the composition of young entering the population varies with the specific timing and location of settlement and whether trait-based selective mortality similarly varies over time and space. Results demonstrate that early life history traits differ with lunar timing of settlement, magnitude of settlement, and settlement habitat, including density of conspecifics. Further, selective mortality interacts with the timing and location of settlement to influence the suite of traits of surviving juveniles.

### Early life history traits vary by lunar phase of settlement

Monthly settlement of *S. partitus* consistently peaked during the summer months, especially in July and August, but there was also substantial interannual variability. These settlement patterns obtained by examining the otoliths of new recruits from monthly surveys and adjusting for mortality paralleled patterns in the supply of settlement stage *S. partitus* to Florida Keys reefs as measured in light traps [8]. For *S. partitus,* there is a linear relationship between the supply of settlement-stage larvae and recruitment in both the Florida Keys [50] and in Barbados [32]. Our 65-mo long settlement record revealed lunar cyclic settlement peaking during the third quarter and new moons, but sufficient settlement also occurred during other phases to enable a comparison of traits across settlement periods. Growth-related traits varied among lunar phases of settlement: Larvae settling to the reef during the dark portion of the lunar cycle, around the third quarter and new moons, were significantly larger and faster growing, and spent less time in the plankton than larvae that settled during the brighter portion of the lunar cycle, around the first quarter and full moons. Thus larvae that settle during the dark half of the lunar cycle are not only potentially less susceptible to visual predation [33], but also arrive with more “optimal” traits [51]. This may be a simple consequence of differential larval growth following spawning. Spawning in *S. partitus* peaks during the dark half of the lunar cycle (third quarter-new moon). Larvae that grow sufficiently rapidly reach a suitable settlement size and are able to settle during the following dark lunar phase (third quarter-new moon). Larvae that do not find sufficient food sources and thus grow more slowly take longer to develop and reach a suitable settlement size, are not ready to settle during the subsequent dark lunar phase, and consequently, settle during the following less optimal light phase (first quarter-full moon). Thus, for larvae settling during the non-optimal light half of the lunar cycle, larval growth is lower, PLDs longer, and settlement sizes smaller. This timing of larval growth and development is likely related to synchrony with larval food sources. In other subtropical environments, zooplankton abundance in the epipelagic layer peaks just before the new moon [52], coinciding with the timing of hatching of numerous species [53], including *S. partitus* [54]. Such an increase in prey items around the new moon could be beneficial for larvae at first feeding as well as just prior to settlement. However, prey are typically patchy and larvae will have differential success in finding food. As noted above, larvae that experience poor feeding conditions and grow more slowly will have to spend longer in the plankton and settle during less optimal times. Protracted delays in settlement have been shown to result in striking declines in larval condition in invertebrate taxa with non-feeding larval stages or specific diet requirements [55], [56], and reduced growth in other fish larvae [57], [58].

Not only did larvae settling during different lunar phases exhibit contrasting traits, but the selective loss of these traits over time also varied among lunar phases. Fish settling during the light lunar phases generally experienced stronger selective loss of individuals with less optimum traits. The primary trait driving reef-based mortality is size-at-settlement [24]. Fish settling during the light half of the lunar cycle were generally smaller and there was stronger selective loss of the smallest settlers relative to those settling during the dark lunar phase. Size-at-settlement is a function of both larval growth and PLD, which are typically inversely related to one another. Thus, light phase settlers experienced selective mortality favoring faster larval growth (associated with larger sizes-at-settlement) and shorter PLDs. Third quarter moon settlers experienced no clear selective mortality related to either of these two traits and selection favored slower larval growth and longer PLDs in new moon settlers. Although fish that are larger at settlement typically have higher survivorship, they spend more time in aggressive territorial interactions and consequently have slower early juvenile growth [24]. Thus settlers during all lunar phases experienced selection against fast early juvenile growth. As a result of different patterns of selective loss of traits across lunar phases, traits of the oldest surviving juveniles (11–28 d old) were generally similar across lunar phases of settlement, except that fish settling during the new moon had significantly faster early juvenile growth. Together these results indicate that the initial composition of settlers interacts with potential lunar-cyclic differences in predation pressure to influence the population composition. In most cases, traits shifted sharply between the larval stage and young recruit stage, suggesting rapid selective loss. Indeed, most post-settlement mortality is thought to occur within the first 2 d of settlement [59]. The relative brightness of the nocturnal reef environment during and immediately following settlement likely influences overall mortality due to predation by visually oriented predators. Nighttime illumination varies predictably over the lunar cycle: regardless of tidal state, the number of moonless hours at night is higher during the period between the third quarter and new moon [8]. Stronger selective loss of early life history traits during the light phases of the lunar cycle is consistent with higher predation pressure by visually oriented predators during and immediately following settlement.

### Early life history traits and relationship to settlement magnitude

While examination of *S. partitus* traits in relation to settlement magnitude across seasonal temperatures indicates that individuals from larger settlement events exhibited faster larval growth than those that settled during small events, this likely simply reflects the general trend of higher recruitment during warm seasons. Controlling for the effect of temperature on growth by examining this relationship *within* the peak settlement season during the warmest months, and limiting the analysis to individuals settling during the darker half of the lunar phase, revealed the opposite pattern. Within the peak settlement season, individuals arriving in the largest pulses during the dark lunar phases had slower larval growth, longer PLDs, and were larger at settlement. As noted earlier, size-at-settlement is the most important trait influencing early survivorship of *S. partitus* [24]. In this case, within the overall warm season when larval growth was already relatively high, the largest sizes-at-settlement were achieved by longer PLDs. Since PLD is inversely related to larval growth [24], somewhat reduced larval growth may simply be a consequence of a significantly longer PLD. The magnitude of *S. partitus* settlement across seasons in San Blas, Panama, was positively related to larval growth [15], but because temperature-related influences on growth were not controlled for, it is hard to resolve whether fast growth was directly related to successful settlement or simply correlated with seasonal patterns of recruitment.

Once settled, *S. partitus* recruits in the Florida Keys did not maintain these differences in traits. Fish that settled in small pulses experienced more trait-based selective loss (i.e., larger difference in traits of larvae and juveniles) than fish that settled in large pulses. Strong selection favoring slower larval growth, longer PLDs, and larger sizes at settlement for fish settling in small events resulted in surviving juveniles from all events exhibiting similar ETHTs. The only exception was that stronger selection for slow early juvenile growth in fish from small pulses resulted in these fish having significantly slower early juvenile growth than juveniles from large events. Thus, overall, fishes settling in small pulses arrived with less optimal traits and consequently, suffered stronger selective mortality which resulted in survivors with a similar range of early life history traits.

It is important to note that we used a cross-sectional approach to compare traits of fishes of different ages to detect patterns in selective mortality. These fish were not from the same cohort, but were from many cohorts integrated over time. There are disadvantages to this method, as neither time of hatching nor settlement was controlled for and individuals may have been exposed to different environmental features that were unmeasured, but contributed to differences in traits with age. However, such cross-sectional approaches have been used before to examine selective mortality processes [60], [61], and in the present study we integrated over a large number of cohorts spread over 6 years, potentially averaging out the noise associated with individual cohort variation. Importantly, the overall patterns of selective mortality we obtained from this approach are consistent with those we obtained from tracking 16 individual cohorts over time [24].

### Spatial patterns in recruitment and early life history traits

Recruitment to offshore bank reefs of the upper Florida Keys was 12-fold higher than to the inshore patch reefs. This may be due, in part, to differences in physical processes that influence larval transport, and depletion of larvae as they cross the reef crest [8], however, there is also evidence that habitat in the inshore patch reefs is less optimal than along the offshore bank reefs for this species [62]. There was a three-fold greater abundance of recruits in coral rubble than on the reef crest and *S. partitus* juveniles have been shown to suffer higher mortality on boulder coral than in coral rubble piles [37], [62]. While there were not enough recruits from the patch reef sites to compare traits of recruits to those habitats with those that settled along the fore reef, we compared growth-related early life history traits among individuals that settled on the reef crest versus surrounding rubble on the offshore bank reefs. Individuals that settled on the reef crest and survived 11–28 d were larger-at-settlement than those in rubble habitats. While 1–10 d old recruits in reef habitats also had higher larval growth rates, these differences disappeared over time. Because habitat-specific traits of settling larvae could not be determined due to the sampling method (light traps), we cannot fully disentangle the relative importance of pre- and post-settlement processes in creating these spatial patterns. For example, differences in larval growth of recruits could have been due to differences in the traits of settling larvae or due to selective processes acting immediately post-settlement (i.e., before the recruits were collected; [63]). However, we can conclude that the magnitude of selective mortality based on settlement size and PLD was larger in the reef habitat. Consistent with habitat-specific abundances and previous mortality estimates [37], selective mortality appears to be stronger in reef habitats.

### Density-dependent effects on recruitment and early life history traits

For the warmest months from 2007–2008, monthly *S. partitus* recruit density was strongly positively correlated with the density of >1 mo. old juvenile conspecifics (2–3 cm SL). This is consistent with a pattern of monthly pulses in settlement following one another, but could also indicate enhanced recruitment as a result of the presence of conspecifics. Further evidence that the presence of conspecifics can enhance recruitment is the positive relationship between recruit density and density of intermediate-sized conspecifics (30–50 mm SL), which should be at least several months old and could have settled during a different season. Presence of conspecifics can enhance reef fish settlement [64], [65] or inhibit it [34], [66]. Interestingly, the relationship between recruit density and adult conspecific density was not significant, and if anything, appeared negative. The presence of conspecifics may signal high quality habitat, but *S. partitus* is a highly territorial species and reproductively mature adults may aggressively deter settlement of potential competitors. Younger conspecifics may not be as disturbed by new settlers or as effective in removing them. While recruitment magnitude was not significantly correlated with adult conspecific density, some early life history traits of recruits were. Larval growth and size-at-settlement were greater for recruits surrounded by higher densities of adult conspecifics. Since these traits are related to enhanced survival [24], it is likely that only the best performers can survive high densities of adult conspecifics (high densities of conspecifics have been associated with elevated mortality of juvenile *S. partitus*; [28], [67]. The presence of conspecifics may initially serve as a cue for settlement, but the aggressive nature of adult *S. partitus* may also prevent all but the strongest recruits from remaining within high densities of adults.

### Conclusions

Results of this study demonstrate that early life history traits interact with both temporal and spatial patterns of settlement to influence reef fish juvenile demography. The distribution of early life history traits of settlers is related to both timing and location of settlement. Post-settlement processes tend to obscure pre-settlement patterns, and in some cases, create new patterns of variation. Individuals settling during optimal times (i.e., dark portion of lunar cycle, larger events) arrive with better traits, such as larger sizes-at-settlement. Large settlement sizes can be achieved by faster larval growth or longer PLD and it appears that both scenarios are used by *S. partitus*. Fish settling during the dark as opposed to the light half of the lunar cycle achieved larger settlement sizes through faster larval growth, but within peak settlement periods, larvae settling during large events achieved larger sizes by spending more days growing in the plankton. Regardless of the mechanism, most of the variation in traits among individuals that settled during different lunar phases and settlement magnitudes was not maintained in the juveniles over time. Selective mortality processes varied in strength and direction depending on lunar phase, resulting in juveniles with rather similar traits.

Early life history traits also varied as a function of settlement habitat. Recruits in reef habitats or within high densities of adult conspecifics were larger at settlement compared to those in rubble habitat or within low densities of adults. Differential selective mortality pressures acting across variable settlement habitats resulted in spatial differences in the distribution of early life history traits. In sum, differences in early life history traits of settling larvae due to timing or magnitude of settlement are further modified over spatial scales depending on habitat and strength of selective mortality processes. Therefore, for *S. partitus*, and probably other species with similar life histories, the specific timing and location of settlement define the composition and demography of the juvenile population.

## Supporting Information

Figure S1 **Map of the upper Florida Keys with sampling sites for light trap deployment and recruit surveys and collection.**
(TIFF)

Figure S2 **Age distribution of** ***Stegastes partitus*** **recruits in the upper Florida Keys (bars) with a function (y = 0.0671x^2^–5.0165x+89.763) fitted to the slope of all recruits >9 d old post-settlement (line) to estimate mortality.**
(TIFF)

Figure S3 **Back-calculated settlement of** ***Stegastes partitus*** **recruits collected in the upper Florida Keys from April 2003 to August 2008, adjusted for recruitment magnitude (density) and mortality, plotted over (upper) a single lunar cycle and (lower) a single maximum tidal amplitude cycle.** Two sample maximum tidal amplitude cycles plotted for representative days when maximum occurred in conjunction with the new moon (solid line) and the full moon (dashed line). New moon indicated by a solid circle and full moon by an open circle. Arrow indicates mean day about which settlement peaked.
(TIFF)

Figure S4 **Back-calculated timing of successful spawning by** ***Stegastes partitus*** **for (a) recruits collected from April 2003 to August 2008, and (b) adjusted for recruitment magnitude (density) and mortality.** (c) Back-calculated timing of successful spawning of settlement-stage larvae collected in light traps from May 2003 to January 2004 plotted over a single lunar cycle.
(TIFF)
