# Supplementary figures and images for: Characteristics of Settling Coral Reef Fish Are Related to Recruitment Timing and Success
Source: PLoS One. 2014 Sep 24;9(9):e108871. doi: 10.1371/journal.pone.0108871 (PMC4177557; doi:10.1371/journal.pone.0108871)

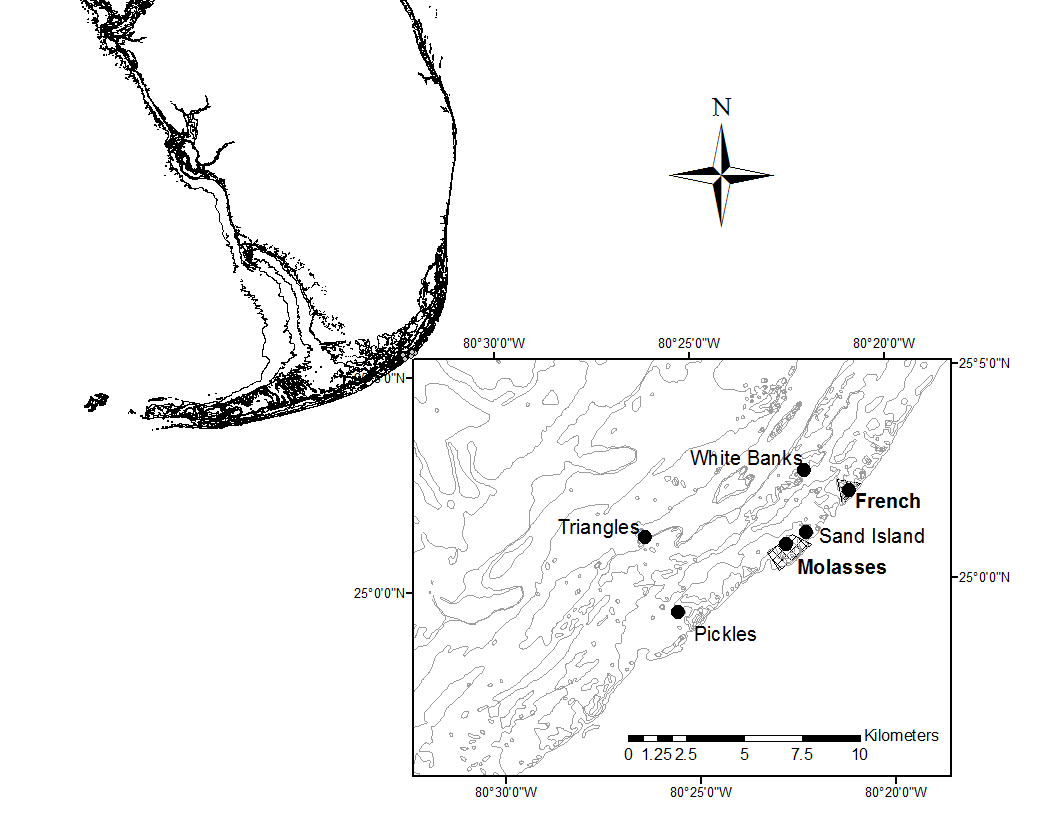

Supplement: Figure S1 — Map of the upper Florida Keys with sampling sites for light trap deployment and recruit surveys and collection. (TIFF) [file pone.0108871.s001.tiff]

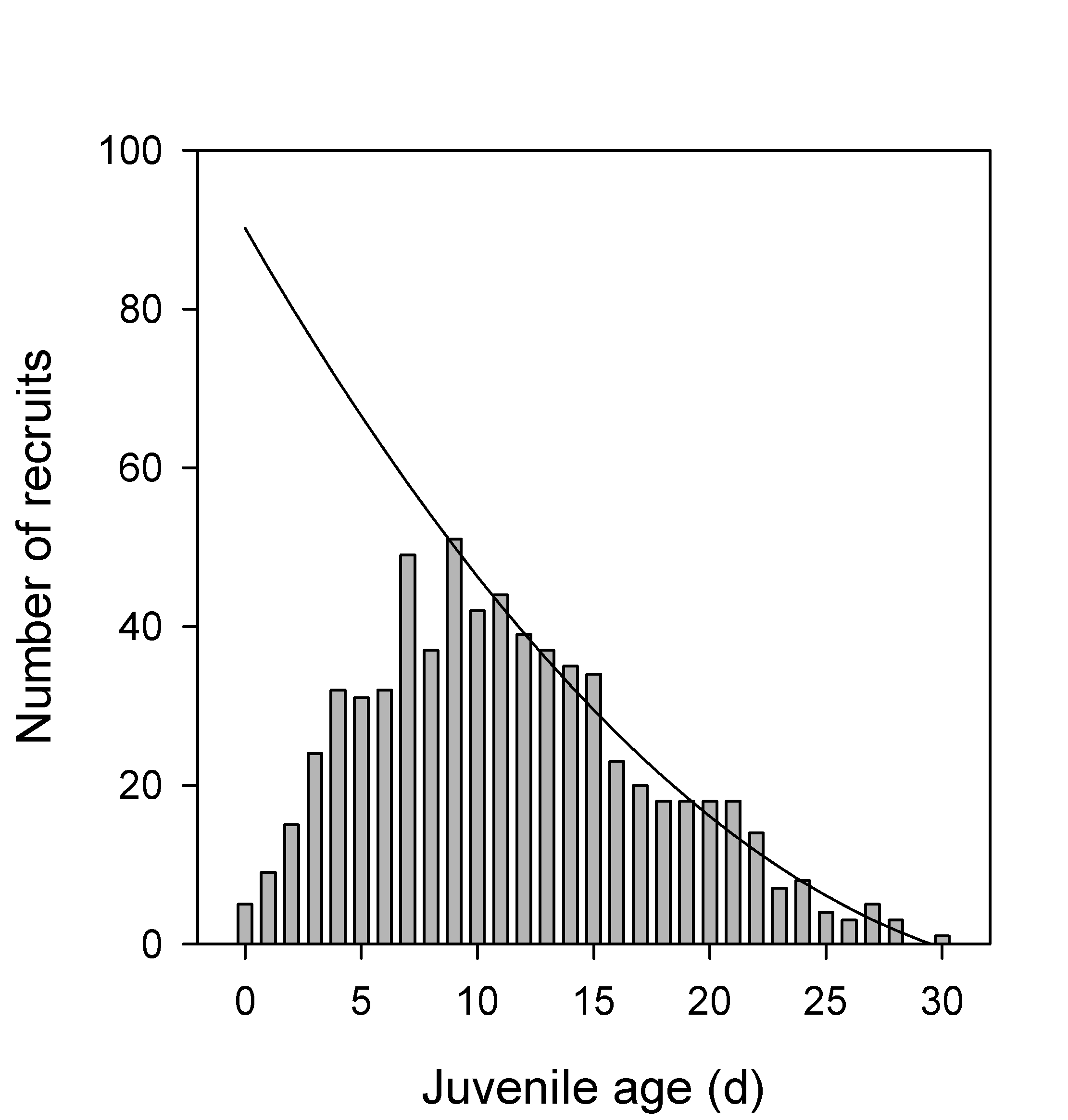

Supplement: Figure S2 — Age distribution of Stegastes partitus recruits in the upper Florida Keys (bars) with a function (y = 0.0671x2–5.0165x+89.763) fitted to the slope of all recruits >9 d old post-settlement (line) to estimate mortality. (TIFF) [file pone.0108871.s002.tiff]

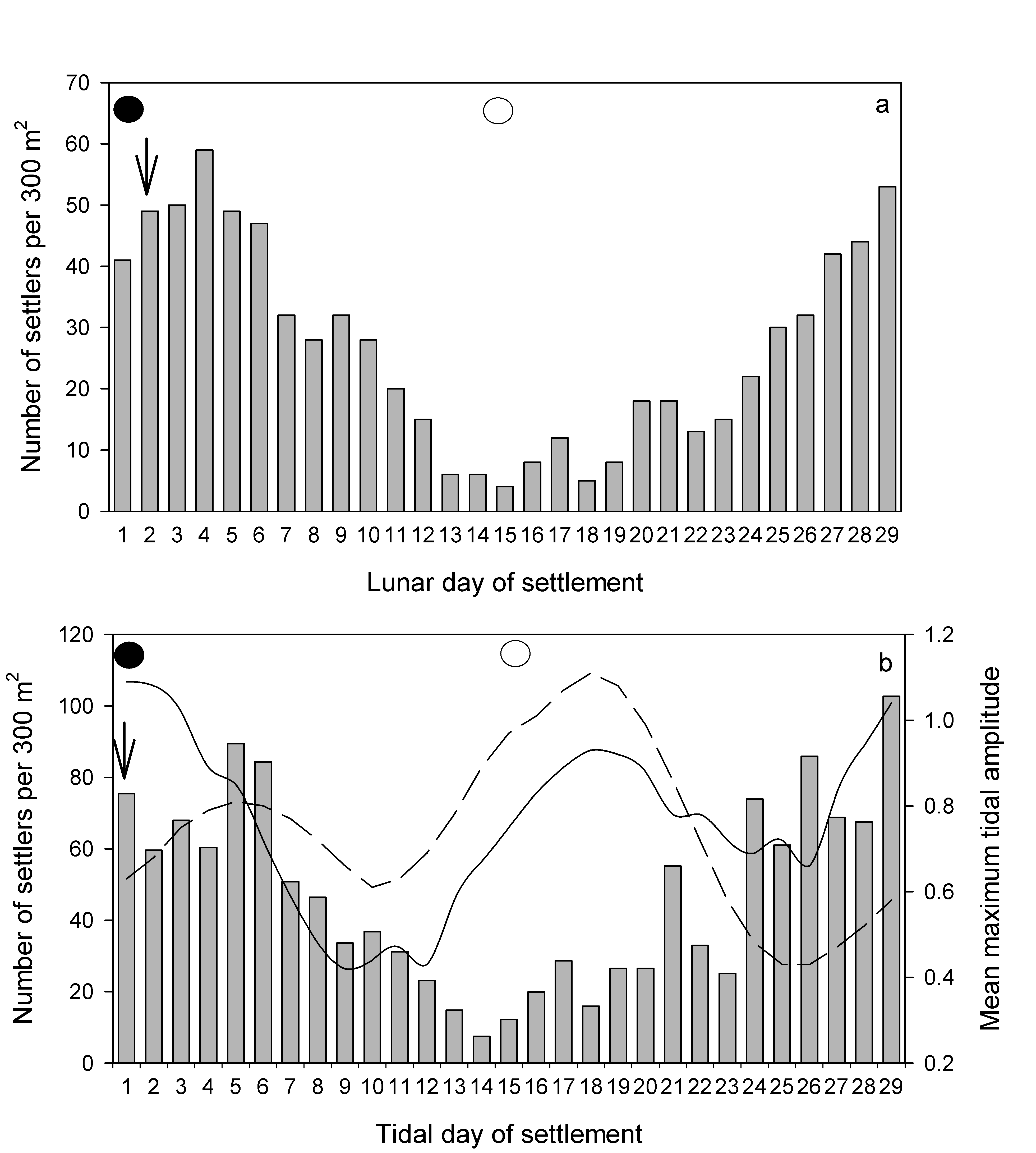

Supplement: Figure S3 — Back-calculated settlement of Stegastes partitus recruits collected in the upper Florida Keys from April 2003 to August 2008, adjusted for recruitment magnitude (density) and mortality, plotted over (upper) a single lunar cycle and (lower) a single maximum tidal amplitude cycle. Two sample maximum tidal amplitude cycles plotted for representative days when maximum occurred in conjunction with the new moon (solid line) and the full moon (dashed line). New moon indicated by a solid circle and full moon by an open circle. Arrow indicates mean day about which settlement peaked. (TIFF) [file pone.0108871.s003.tiff]

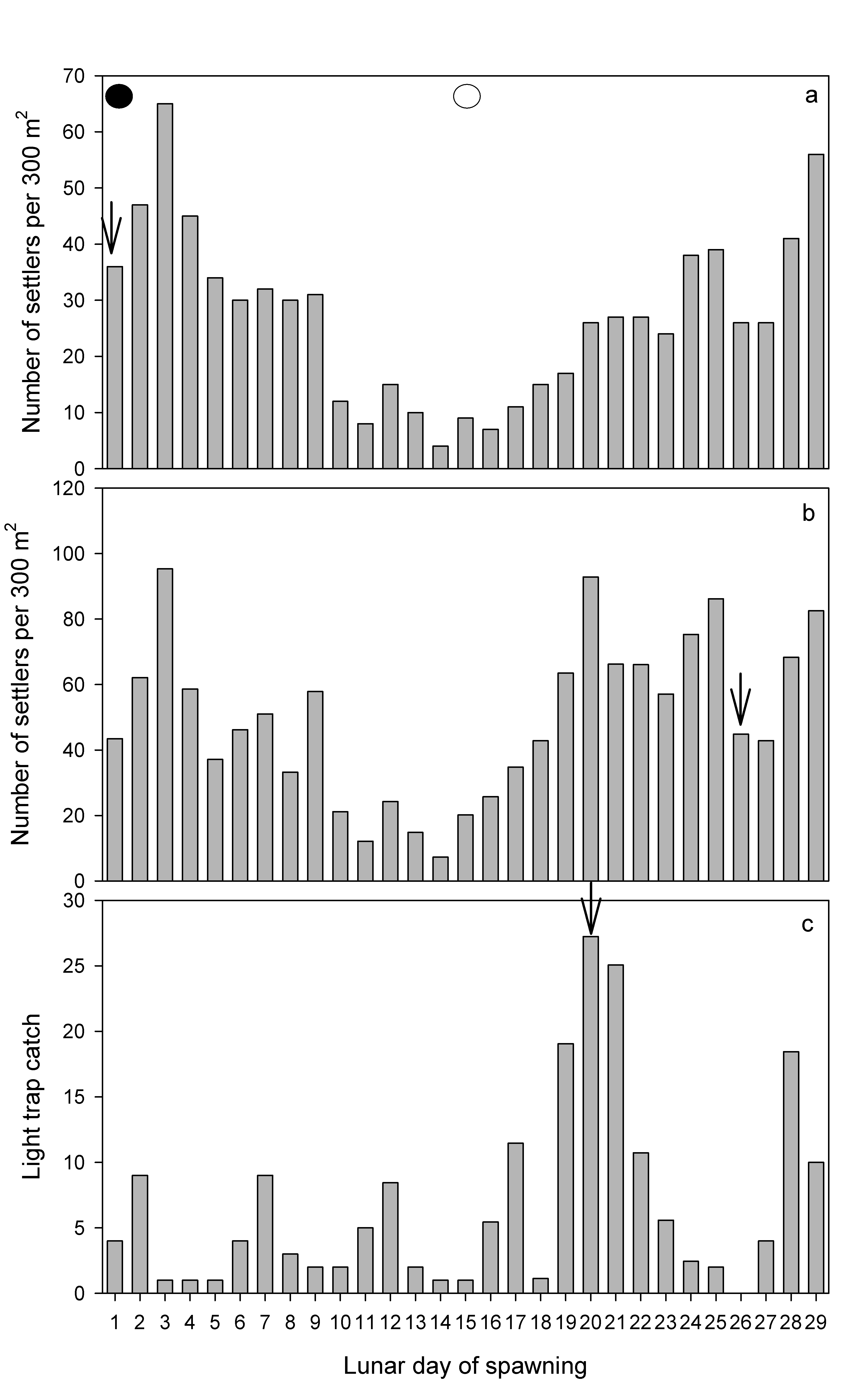

Supplement: Figure S4 — Back-calculated timing of successful spawning by Stegastes partitus for (a) recruits collected from April 2003 to August 2008, and (b) adjusted for recruitment magnitude (density) and mortality. (c) Back-calculated timing of successful spawning of settlement-stage larvae collected in light traps from May 2003 to January 2004 plotted over a single lunar cycle. (TIFF) [file pone.0108871.s004.tiff]
